# Adoption of improved cook stoves by households in informal settlements of *Woreda 12*, Yeka subcity, Addis Ababa

**DOI:** 10.1186/s13705-022-00370-4

**Published:** 2022-11-07

**Authors:** Nibretu Kebede, Degefa Tolossa, Tamirat Tefera

**Affiliations:** 1 Center for Environment and Development, College of Development Studies, Addis Ababa University, Addis Ababa, Ethiopia; 2 Geography and Development Studies, Addis Ababa University, Addis Ababa, Ethiopia

**Keywords:** Informal settlement, ICS, *Lakech* stove, *Mirt* stove, Three-stone stove, Addis Ababa

## Abstract

**Background:**

This study analyzed the factors affecting the use of improved cook stoves (ICS) in informal settlements of Addis Ababa based on the data generated from 450 households drawn from *Woreda* (*Woreda* is a local term used to describe the lowest administrative unit of Addis Ababa City Administration, Ethiopia.) 12 of *Yeka subcity.* It examined the interactive effect of households’ socio-economic backgrounds and energy sources on the adoption of ICS. The data were analyzed using descriptive methods and the multinomial logit model.

**Results:**

Demographic and economic factors such as sex of the household head,[Household head is the one who has an income and decision-making power in family affairs (a husband for married people)], family size and family income have no relationships with households’ ICS use while education level, number of years lived in the area, type of home owned, and stove-operating costs have a significant influence on the choice of an ICS. Households that live in a good home (made from wood and cement) used more *Mirt* (*Mirt* is an improved firewood stove mainly used to bake *Injera* and bread.) and *Lakech* [*Lakech* also called *Tikikil* is an improved charcoal stove used to cook different kinds of dishes (non-*Injera*)] stoves than the traditional three-stone stoves. On the other hand, household heads with higher levels of education and who have lived more than 7 years in the area in a better home owned more ICS than the traditional three-stone stoves.

**Conclusions:**

The availability, affordability, durability and simplicity to operate stoves, and subsidies affect the choice of an ICS. Energy sources that are commonly used by households in informal settlements also have a strong influence on the choice of energy-efficient stoves. Compared to ICS, heavy use of traditional three-stone stoves by households that already have access to electricity, directs government policies to focus on providing reliable electric service and subsidize those using ICS.

## Background

Energy-efficient cooking technologies or simply ICS increase energy efficiency [1], reduce heavy reliance on fuel wood and energy consumption levels, and save cooking time [2, 3]. They improve the taste and flavor of food and the quality of life [4–6]. Likewise, they help to manage demand and save energy by replacing conventional energy technologies [7], efficiently convert biomass into energy, emit very little smoke associated with complete combustion, and contribute to the sustainable use of scarce resources [8–10]. Switching to modern stoves and fuels and using ICS with better technological designs transforms lives by improving health, protecting climate and environment, empowering women, lowering households’ expenses on medicines; reducing the workloads of women and children; and improving flexibility for women with regard to labor, time and money [11–13].

Projections for 2030 indicated that aggressive energy efficiency measures can significantly reduce the demand for energy. For instance, a study conducted by Reyna and Chester [1] indicated that the adoption of light-emitting diodes (LEDs) reduces residential electricity consumption for lighting by 53%, and that upgrading electrical appliances could lower it by 28%, and replacing light bulbs and incandescent lamps with more efficient compact fluorescent lamps could lower residential electricity consumption for lighting by 75%, ICS reduce firewood consumption by 40–60% and charcoal use by 30–40% compared to traditional stoves [11, 14, 15]. Such energy-efficient technologies can produce the same amount of light with less energy and expense of firewood whereas traditional stoves are likely to break easily when moved from place to place [6, 16]. High storage capacity batteries and solar panels are also useful, resilient and clean energy sources used in all geographical areas [15, 17].

The multi-tier framework (MTF) developed by the World Bank provided a comprehensive guide to analyze the factors affecting households’ choice of energy-saving stoves. These factors are availability of energy sources, uninterrupted power supply, durability and affordability of the stove, price of fuel, the capacity to load big appliances, the health and safety of using the stove, and the formality of the service provided [18]. In line with this, Yonas, Abebe, Köhlin, and Alem [19] found that as income increases, households are more likely to buy ICS that lower energy cost and use clean energy sources. Medina, Cámara, and Monrobel [20] and Amoah [21] also suggested that substituting declining biomass, adopting energy-efficient stoves and changing households’ energy consumption behaviors are effective tools to achieve economic outcomes and lower environmental pollution.

However, studies indicated that limited supply and high cost of energy-efficient stoves at local levels, shortages of electric meters, unavailability and high cost of spare parts, lack of access to credit facilities, and spending priorities for other basic needs affect the use of energy-efficient technologies [22–27]. The cost of fulfilling ICS and cooking appliances is capital intensive and unaffordable. Padam et al. [18] underlined the importance of providing incentives and arranging flexible payment systems and credit facilities. A study compiled by Mfundis and Commeh [7] indicated that socio-cultural, behavioral and competence-related barriers also make the adoption of ICS technology socially unacceptable at the household level. For example, youth in Botswana have made collecting fuel wood and making fire part of their culture. Some of them do not even recognize the indoor air pollution effects of traditional stoves, firewood savings and the health benefits of using ICS [28, 29]. Feldmann and Otremba [15] and Chagunda, Kamunda, Mlatho, Mikeka, and Palamuleni [3] described that households prefer to use traditional three-stone stoves over power-saving technologies whilst food such as *Injera*^1^ baked on an ICS does not smell smoky, has good taste, and the edges are smooth [30].

48.27% of the total population has access to electricity in Ethiopia, with a current production of 4284 MW, where the domestic energy consumption amounted to 92% of the energy supply, where waste and biomass are the primary sources of energy amounting to 92.4% of Ethiopia’s energy supply, where 84% of urban households used biomass, and 63.3% used traditional three-stone stoves as their primary stove [18, 30–33]. The use of stoves such as flat *Mitad*^2^ and *Fermelo*^3^ requires low initial cost, which is highly consistent with consumers’ preferences, i.e., ease of use and relatively wide availability [25]. The cost of cooking appliances such as kittles, flat *Mitad* and pots are cheaper and the technology is more easily adaptable than electrical items such as electric ovens, toasters and water boilers. Culturally, people like to see open fire and be around it, smoke makes food smell nice, and women like to go out collecting fuel wood as it gives them a space to socially interact [7]. Traditional three-stone stoves can be easily set up, fit all pot sizes, the heat from the fire provides warmth, light, a sense of comfort and the use of biomass is often the only available, accessible and affordable fuel for most households [15]. On the other hand, traditional open fire cook stoves are not fixed in one place and when these stoves move from place to place, the risk of breakage is high.

However, heavy reliance and inefficient usage of biomass in open fire burning wastes resources and overconsumption of biomass causes many harmful impacts that impede economic and social development in developing countries [13, 34, 35]. According to WHO, inefficient combustion of solid fuels in low-quality open fire and outdated stoves, operated in poorly ventilated kitchens and excessive exposure to smoke impact the health of women and children [12, 18]. It causes 4 million deaths and produces 1 gigaton of CO_2_ emissions every year from burning wood fuels [11]. Globally, it resulted in severe respiratory diseases responsible for up to 12% of deaths in Ethiopia, makes clothes dirty and smells like smoke, irritates eyes and creates a sense of discomfort [30, 36]. Burning biomass in such stoves has undesirable consequences to the environment such as the decline in the availability of biomass resources, deforestation, and greenhouse gas emissions contributing to climate change. Most studies indicated that households will continue to depend on these fuels for decades to come [30].

Whilst ensuring access to affordable, reliable, sustainable, clean, safe, healthy and modern energy for all by 2030 is one of the 17 Sustainable Development Goals of the UN, only 4.1% of households in Ethiopia use electricity as a primary cooking fuel [18]. Energy sources such as biogas and Liquefied Petroleum Gas (LPG) are rare, accounting for less than 1% of households and are unaffordable [18]. Similarly, although solar energy is easily available, clean, and very effective to prepare staple foods, generating electricity using these sources is a recent practice in Ethiopia. Beyond this, not all traditional dishes can be prepared with solar energy, and solar cookers are also expensive to use. As a result, the government has prioritized the use of ICS and efficient lighting as the most important areas to guide investments, expand energy supply, and ensure environmental sustainability [37].

Classification of ICS based on fuel use, portability in the kitchen and different sizes is also essential in the choice of stoves [15, 35]. For example, *Mirt* stoves using firewood to bake *Injera,* are designed to reduce environmental degradation whereas *Lakech* stoves use charcoals. Disregarding the facts that the performances of these stoves are evaluated mainly by the amount of generated energy which is absorbed by the cooking pot (heat-transfer efficiency) and the amount of generated energy which is converted to heat, as well as the amount of carbon dioxide which is developed (combustion efficiency) [38]*,* household members use these stoves for preparing different kinds of foodstuffs.

Lack of access to legal land entailment forces households to construct houses below the standard and becomes the major impediment to get access to electricity. This in turn forces many households to use open fire traditional three-stone stoves while wealthy and educated households are likely to adopt energy-efficient stoves. Many studies focused on alternative energy sources, the available technologies and the deteriorating living conditions of households in rural and urban areas of Ethiopia [19, 39–41]. However, in Ethiopia, little has been done for the adoption of energy-efficient stoves in informal settlements.

This study addressed the questions: (1) What kind of stoves households own in informal settlements? (2) What factors attribute to the adoption of ICS? (3) How energy sources and households’ socio-economic backgrounds affect the adoption of ICS? The study considered households’ socio-economic and demographic characteristics, financing options such as credit facilities and subsidies affecting the choice of energy-efficient stoves.

The study is set in four sections: the first section provides the background of the study followed by a section that explains the data and methods used. The second section begins with a description of the study area, how the sample is designed, and then data collection and analysis methods employed follow. The third part presents the results of the study and discusses the findings. The final section provides conclusions, implications and future research directions in the area.

## Data and methods

### Description of the study area

**Fig. 1:**
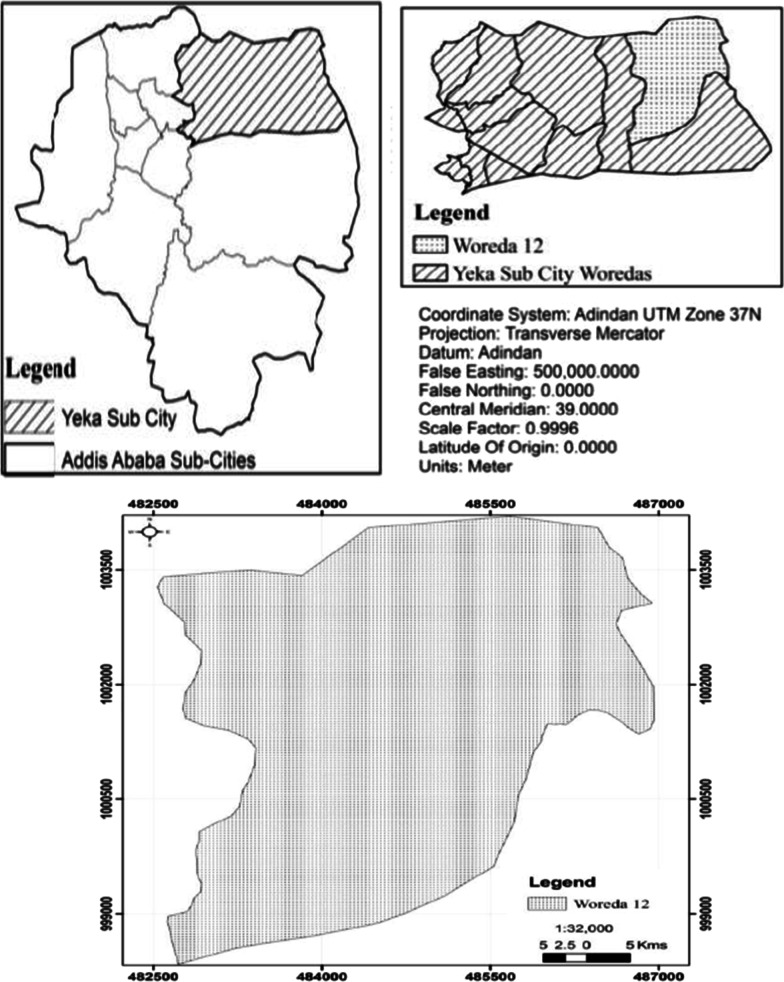
Location map of the study area. Source: modified from EthioGIS shape file

Like other developing cities, the city of Addis Ababa is faced with multiple development challenges. The city is expanding in a sprawling manner, and around 30% of the population lives under poor living conditions [42]. It has ten subcities and four of them are found in the down town of the city while the rest border rural areas (Fig. 1). Among the six subcities found in the outreach areas of Addis Ababa, informal settlers in Yeka subcity are settled in ragged areas, live close to forests and the subcity borders are far from the surrounding region. This situation has caused researchers to focus on this subcity.

*Woreda* 12 is one of the 13 districts in Yeka subcity that shares the largest territory with the cultivated edge of rural areas. It is located at about 9°3′2″N, 38°52′41″E, 2450 m above sea level, is found approximately 11 km from the city center and is situated around the holy church of *Kotebe Gabriel and Kotebe* Metropolitan University. Based on the data compiled from surveys conducted in the study area, 78% of informal settlers have access to roads and transportation, 80% to education and health centers, 20% live around river banks and low laying areas, 47% live close to forest resources and 38% are located in a rugged topography. Informal settlers are in particular located in *Kotebe Gebriel, Hibret Amba, Rediet, Happy Village, Mesalemia, Sara Park, Kara and Demamit* sites.

### Sample design and sampling method

Considering the existence of a very large number of informal settlers and their similarity on the one hand, and the difficulty to cover all sites in a limited time and financial constraints, on the other, have forced the researchers to down-scale the sample design to the household level and subdivided respondents in three stages.

*First* Yeka subcity was purposively selected among the ten subcities in Addis Ababa and *Woreda* 12 was purposively selected among the 13 districts in Yeka subcity due to its location in the expansion/outreach areas and longest territory which it shares with a neighboring region relative to the other districts in the subcity.

*Second* In *Woreda* 12, there are eight sites and 2,590 informal settlers officially registered by the local administration. From these households, 1926 are electric users^4^ found in seven sites and 664 are non-users^5^ of electricity found in three sites. This number is still very large and researchers cannot cover all of them. Instead, it served as a sample frame to select specific study sites and respondents using purposive and random sampling strategies, respectively. As a result, two sites from electric users (that is, *Kotebe Gebriel and Hibret Amba* with a total of 576 informal settlers) and two sites from non-users of electricity (that is, Kotebe Gebriel and Demamit with 516 informal settlers) were selected purposively. This helped to develop a balanced sample frame to choose electric users and non-users of electricity.

*Finally* Once the sample frame is specified, the representative sample sizes for the study were drawn randomly considering the relative heterogeneity among the sites, and the relative homogeneity among households within the same site was determined with a 95% confidence interval and 450 sampling units (comprising 229 electric users and 221 non-users of electricity). However, due to a lack of legal living status of informal settlers, strict randomization was not possible to select households for the study. To minimize the effects of this problem, respondents were selected using a proportional sampling method that gives equal opportunity to each household in both electric users and non-users of electricity groups.

### Data sources and analysis methods

Primary data were obtained using a multi-tier questionnaire that helped to capture information about households’ energy sources and factors affecting energy choice. The questionnaire was structured to cover households’ socio-economic characteristics, food consumption behavior, energy-saving stoves owned, the reasons for adopting the stoves that are currently owned, the adoption level of ICS and the challenges encountered in using energy-efficient technologies. The questionnaire was administered to 450 randomly drawn households found in Kotebe Gebriel, Hibret Amba and Demamit representing 2690 informal settlers. The survey was managed by the researcher and properly selected, well trained and closely supervised enumerators. The list of informal settlers that served as a sample frame was obtained from the computerized data base of *Woreda* 12 Administration.

Field work during the pilot study and data gathering stages helped to observe the housing conditions, the landscapes, availability of infrastructures in the study area and to closely monitor the activities of data collectors. To minimize distortions and personal biases associated with respondents’ opinions and attitudes, the validity and reliability of the data gathered was verified carefully using statistical software. However, due to a lack of legal living status of informal settlers, strict randomization was not possible and some settlers were even reluctant to fill in the questionnaire or unavailable during surveys. Those problems were managed and the margin of errors was minimized by substituting them by others using the nearest neighborhood approach.

Descriptive statistics, such as frequency tables, percentages, bar graphs and figures, were used to present and analyze the influence of demographic variables affecting ICS use, to explain the relationship between the duration of ICS adopted by households and fuel consumption trends, to discuss alternative methods of financing energy-efficient stoves and factors affecting the adoption of ICS, to explain the conditions to use solar and electric stoves, and to present the rationale for using ICS and to describe the major problems that households in informal settlements encountered.

The *multinomial logit model* was used to analyze the adoption of energy-efficient cooking stoves in informal settlements*.* It combined a set of factors affecting the choice of energy-efficient stoves. To analyze the determinants of ICS use, the dependent variable is households’ alternative cook stoves (the traditional three-stone stove, *Mirt, Lakech* and electric stoves) and explanatory variables are developed based on the theoretical frameworks and expected to influence households’ usage of energy-efficient stoves. The model that integrates these variables and helps to interpret the effects of a set of explanatory variables with regard to the adoption of energy-efficient technologies is provided as follows:

$${Y}_{i}={\delta }_{i}+{\beta }_{1}{X}_{1}+{\beta }_{2}{X}_{2}+\dots +{\varepsilon }_{i},$$

where ${Y}_{i}$ = the outcome variable for a household adopting energy-efficient technologies (such as power-saving electric stoves and improved biomass stoves like *Lackech and Mirt*); ${X}_{i}$ are explanatory variables that includes demographic, economic and stove-related factors; ${\delta }_{i} and {\beta }_{i}$= parameter estimates; ${\varepsilon }_{i}=$ error terms.

## Results and discussion

### Factors affecting energy-efficient stove use: descriptive analysis

Table 1 provides the stoves owned by households based on their socio-economic profiles. The order of these stoves is presented based on their energy efficiency level. As provided by Yonas and et al. [19] and Zenebe et al. [43], this order spans from the traditional three-stone stoves with the lowest technology level through improved biomass stoves (*Mirt*) and charcoal stoves *(Lakech)* to electrical stoves with advanced technology.

**Table 1:** Stoves owned by households based on socio-economic backgrounds (%)

Demographic and socio-economic factors		3-stone stove	Mirt/Lakech stove	Electric stove	Total
Sex	Male Female Total	13 7 20	29 13 42	21 17 38	63 37 100
Family size	Below 3 families 3–4 families More than 4 Total	3 8 9 20	4 22 15 41	5 24 10 39	12 54 34 100
Marital status	Not married Married Separated Total	4 15 1 20	11 28 2 41	3 35 1 39	18 78 4 100
Education	Below grade 4 Grade 4–8 Grade 9-Diploma Degree and above Total	3 6 8 2 20	6 11 14 10 41	0 4 8 27 39	10 21 30 40 100
Family income per month	Up to 6000 Birr* Above 6000 Birr Total	7 13 20	16 25 41	4 35 39	27 73 100
Years lived in the area	Up to 3 years 4–6 years 7–9 years Above 9 years Total	5 6 3 5 20	9 11 11 10 41	2 8 9 20 39	16 26 23 35 100
Home type /condition/	Poor (wood and mud) Good (wood and cement) Very good (steel and blocket) Total	9 10 0 19	11 30 1 42	4 30 5 39	24 70 6 100
Number of dwellings	1–2 rooms Three rooms More than 3 rooms Total	12 4 3 20	18 15 10 42	7 13 18 38	37 31 31 100

Source: Survey data, March 2022. *Birr is the local currency of Ethiopia. Its official exchange rate in February 2022 was 1 USD = 51 *Birr*

The survey result indicated that 20% of households in informal settlements used open fire traditional three-stone stoves, 42% *Mirt/Lakech* stoves and 38% electric stoves as their primary cooking stove. Male and female headed households considered in this study are 63% and 37%, respectively. About 13% of male headed households and 7% of female headed households used traditional three-stone stoves; 29% males and 13% females used *Mirt/Lakech* stoves; and 21% males and 17% females used electric stoves. However, although the number of males is greater than that of females in all cases, it is difficult to exhibit a clear relationship between sex of the household head and the adoption of energy-efficient stoves.

In terms of family size, the number of households using traditional three-stone stoves has increased from 3% (below 3 families) to 9% (above four families) whilst the number of households using ICS *(Mirt/Lakech* and electric stoves) varies considerably with an increase in family size. This indicates that family size does not affect the households efficient stove use. However, studies conducted by Bekere and Megerssa [44], Geddafa, Melka, and Sime [45] and Woubishet [46] indicated that sex of household head and family size are important determinants in adopting *Mirt* stoves and biogas technology. In fact, the findings of Bekere and Megerssa [44], Geddafa, Melka, and Sime [45], in particular, are based on the adoption of biogas technology and *Mirt* stoves in rural areas, but not in informal settlements.

Among married household members that constitute 78% of all household members, 35% used electric stoves and 28% *Mirt/Lakech* stoves. This shows that married people are more likely to use ICS than singles. Likewise, higher levels of education of the household head and longer living periods in the area have a positive influence on the adoption of ICS. Income wise, from households earning below 6000 *birr*/month, 7% use traditional three-stone stoves and 20% ICS. From those earning above 6000 *birr*/month, 13% use traditional three-stone stoves and 60% ICS. In both cases, a significant number of households preferred to use ICS indicating that the relationship between family income and stove choice is not clearly described while Sheng, He and Guo [34] and Woubishet [46] concluded that wealth and income are important determinants to adopt ICS.

Informal settlers with existing access to electricity were expected to use electric stoves. But only 73% were found using electric stoves and the rest used traditional three-stone stoves and ICS. From electric users, 72% were found using *Mirt or Lakech* stoves mainly due to a lack of adequate and reliable electric supply.

The relationship between home conditions (described by the type of home owned) and number of dwellings owned and energy-saving stoves adopted by the households is also clearly depicted in Table 1. Among households living in a good house (made of mud and cement) only 10% used traditional three-stone stoves while 60% used *Mirt/Lakech* and electric stoves. From households living in very good houses (made of steel and blockets), households using traditional three-stone stoves is literally nil while those using ICS are 6%. Similarly, among households who live in 1–2 rooms, 12% used the traditional three-stone stoves and 25% used ICS. From those who owned more than 2 rooms, only 7% used traditional three-stone stoves while 55% used *Mirt/Lakech* and electric stoves. These results suggest that home conditions and number of rooms in a home determine households ICS use and as the number of rooms and the conditions of houses owned by households improves, the tendency of those households using energy-efficient stoves increase. This finding is similar to that reported in [46].

**Fig. 2:**
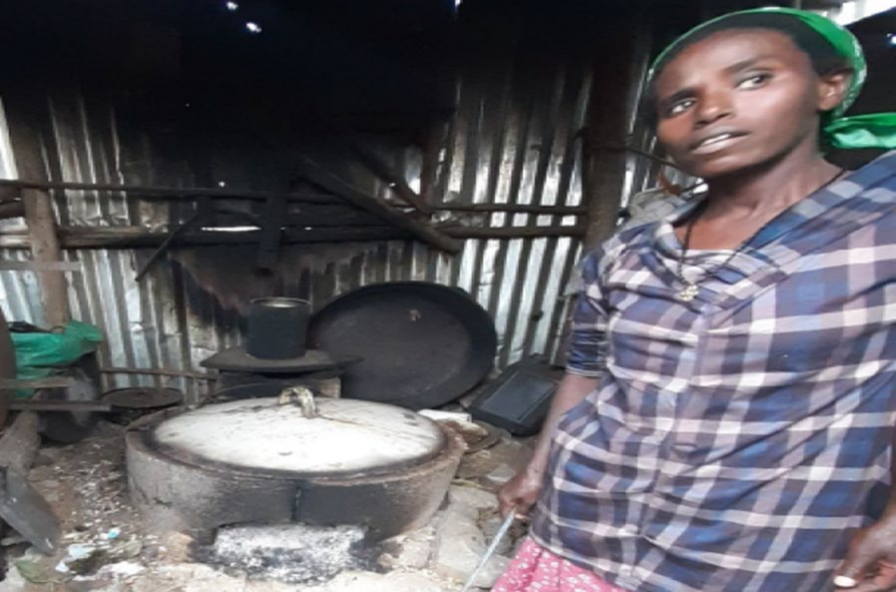
Typical *Mirt* stove owned by households in informal settlements. Source: photograph taken by one of the authors, April, 2020

Figure 2 presents the typical *Mirt* stove used by households in Ethiopia to bake *Injera* and bread. Compared to the traditional three-stone stove tripod, it saves fuel wood from 22 to 31% [41].

In Ethiopia, ICS were primarily designed to solve deforestation problems and pollution effects. They are keys to safe, reliable, affordable and sustainable energy in the future and balance energy scarcity [47, 48]. However, in this study, 50% of households used these stoves to reduce the operational cost of energy by efficiently utilizing biomass and reducing wastages, 31% to save time and reduce workloads of family members involved in cooking activities, and 18% to protect forests and sustainably use scarce resources. This is in line with the research results described by Abebe & Koch [35] and Dawit [49] who emphasized households’ economic reasons to own energy-efficient cooking stoves and shift to renewable energy sources.

Households were required to rate the salient factors that affect their choice of stove based on the most pressing reasons being provided to them. Table 2 presents the five most important factors that influenced households’ decision to own a specific stove. Based on this data, 82% households use traditional three-stone stoves influenced by the availability and price of the stove, 79% prefer to use *Mirt* stoves due to the availability of subsidies, credit facilities, quality/durability and efficiency reasons; and 69% owned *Lakech* due to its capacity to lower energy cost, durability of the stove, the availability of subsidies and credit facilities. The choice of electric stoves depends mainly on cleanness, contribution to minimize indoor air pollution, capacity to save family labor and time, and simplicity and convenience to use the stove technology as confirmed by 68% of households. In relation to this, Feldmann & Otremba [15] concluded that availability, affordability and reliability of fuels and the contribution of stoves to clean burning, the purchasing price of the stove and simplicity to use determines the choice of ICS.

**Table 2:** Key factors affecting households’ stove choice

Stove type	Factors affecting stove choice	Percent
Three-stone stove	1. Cheap technology to buy	43
	2. Widely available stove	39
	3. Well-known stove	8
	4. Lowers the cost of energy	6
	5. Simple/easy to use	4
Mirt stove	1. Most subsidized and easy to obtain credit	33
	2. Quality and durability of stove	25
	3. Lowers the cost of energy	21
	4. Well-known stove	11
	5. Widely available stove	10
Lakech stove	1. Lowers the cost of energy	25
	2. Quality and durability of stove	25
	3. Most subsidized and easy to get credit	19
	4. Widely available stove	16
	5. Cheap technology to buy	16
Electric stove	1. Clean and health source	24
	2. Saves family labor and time	23
	3. Convenient and easy to use	21
	4. Well-known stove	18
	5. Quality and durability of stove	14

Source: Survey data, April 2020

### Duration of ICS adopted by households and fuel consumption trends

**Fig. 3:**
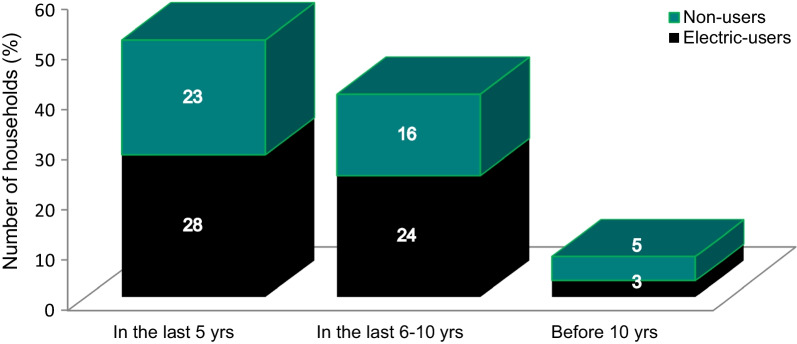
Period improved cook stoves owned by households. Source: Data Developed by the Authors, March, 2022

To better understand the adoption rate of ICS in informal settlements, data are captured from 390 households. Based on this data, 55% are electric users and 45% are non-users of electricity. Depending on the source of energy, about 87% of households owned only one type of stove and others owned two or more kinds of cooking stoves. Figure 3 presents the time when households owned energy-saving stoves (that is, 8% owned before 10 years, 40% before 5–10 years, and 51% in the past 5 years). Based on this data, the adoption of ICS increased greatly in recent periods and the rate of adoption of electric users is greater than that of non-users. As corroborated by Abebe & Koch [33], the adoption speed of ICS also increased with the increased income and varies across regions.

It is also essential to understand the perception of households on fuel consumption trends and the forces that drive them to adopt ICS. The result showed that both electricity and biomass users contend that their consumption levels either remained the same as before or increased over time. Specifically, 22% of biomass users and 42% of electric users perceive that energy consumption has grown significantly whilst 21% of biomass users and 9% of electric users contend that there is no change in energy use over time. In both cases, households are either forced to expend extra cost for energy sources or use ICS with the intent of reducing operation costs. Biomass users in particular should adopt ICS use and focus on shifting to modern energy sources.

### Financing sources and challenges to use energy-efficient stoves

**Fig. 4:**
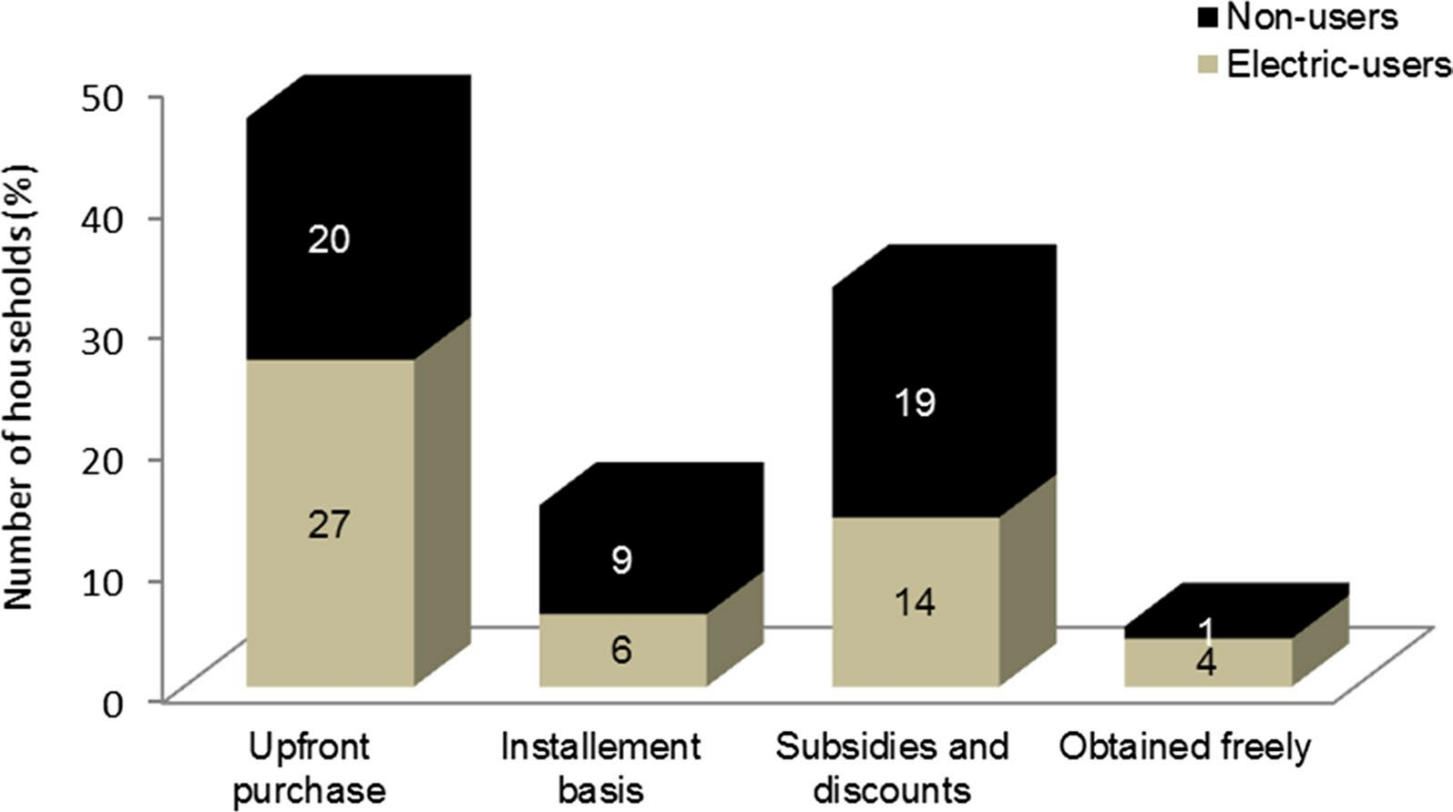
Sources of financing energy-saving stoves. Source: Data Developed by the Authors, March, 2022

Households have alternative financing methods to own energy-efficient stoves. In the study area, about 47% of stoves are owned from own source through upfront payment, 32% were obtained through subsidies and discounted sales, 15% through suppliers’ credit and loans from creditors, and 6% freely and a mix of financing options (Fig. 4). The sources of finance also vary based on household’s electric use status. For example, 27% of electric users obtained a stove via own sources and 6% through an installment basis while this figure amounted to 20% and 9%, respectively, for non-users of electricity. This indicates that unless special arrangements such as subsidies and discounts, incentives and credit facilities should be provided to the low-income households, as they cannot afford to pay the upfront cost of energy-efficient stoves like cylinders, electric stoves and expensive electrical appliances.

Many households believe that subsidies and credit facilities help them to install energy-efficient stoves and enjoy the economic and health benefits of switching to clean energy sources. They also contend that the risk of unreliable electricity supply urges them to own more than one kind of stoves and adopt an energy-stacking approach. On the contrary, a not very low number of households (about 30%) believed that the removal of subsidies, could result in the increase of energy prices, and urging polluters to pay for their pollution effects, encourage households to use energy-efficient cooking stoves and discourage the consumption of traditional energy sources. The risk of unreliable electricity supply also urges households to own more than one kind of stove and adopt an energy-stacking approach.

However, although 38% of households described that they had no problems in using energy-efficient stoves, 62% believed that they faced different challenges (Table 3). Specifically, about 22% were faced with the high cost of obtaining ICS, 12% encountered a lack of maintenance service, 12% described that the stoves are poor in quality, do not last long and are poor in workmanship, and 6% explained that the stoves could not power large appliances. These problems shall require the attention of ICS suppliers, creditors, donors and government bodies. According to Wassie & Adaramola [50], the provision of poor-quality, high cost of solar PV systems, lack of after-sales service, and limited access to credit facilities are critical problems in applying ICS.

**Table 3:** Households’ problems to use ICS

Problems encountered to use ICS	Freq	%
1.No problem encountered till now	172	38
2. Expensive to buy	96	22
3. Absence of maintenance service	55	12
4. Not durable /poor in quality stoves/	53	12
5. Cannot power large appliances	27	6
6. Other reasons	47	10
Total	450	100

Source: Survey data, Feb., 2022

### The use of solar energy and electrical appliances

In urban Ethiopia, the usage of solar energy for domestic application is a recent practice and the least utilized resource. According to Hailu and Kumsa [51], the country uses off-grid solar photovoltaic (PVs) technologies such as distance-education through radios and vaccine fridges in remote areas. Where other fuels are not easily available, it is a useful source of energy for lighting and preparing staple foods. However, the current energy mix in Ethiopia is dominated by hydropower. Projections indicate that this mix will shift to solar and wind energy towards the end of 2050 as a least-cost energy supply option [52]. In rural Ethiopia, electricity has reduced kerosene use, health damages, CO_2_ emissions and helped micro enterprises to generate more business income [50].

**Fig. 5:**
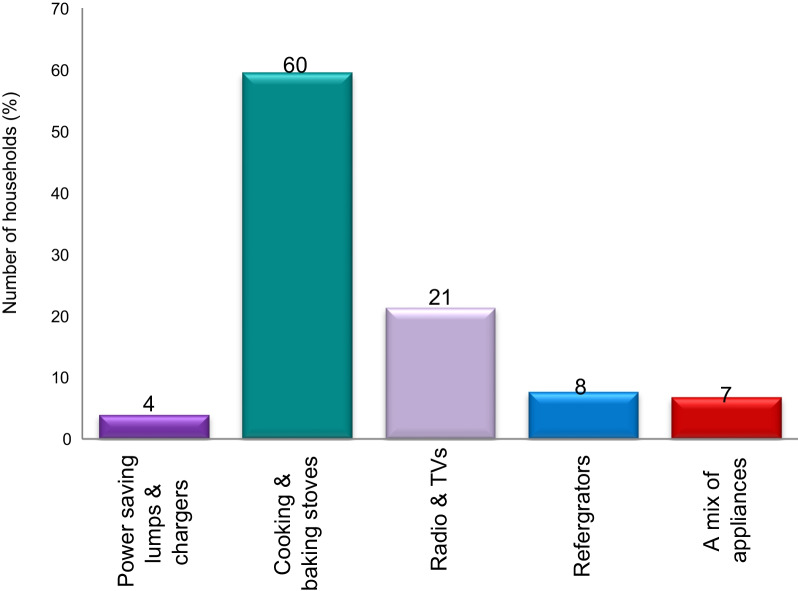
Priority choice of electrical appliances by electric users. Source: Authors own data

In this study, 60% of households used solar energy exclusively for lighting and charging batteries. Solar cookers are too expensive, not widely available, and influenced by cultural factors. On the other hand, traditionally, all dishes cannot be prepared with it. That is, solar energy does not completely replace traditional fuels and stoves [15]. All these facts have forced households in informal settlements to prioritize electrical appliances according to their income level and importance of appliances for a household (Fig. 5). In view of this, 60% want to own baking and cooking devices, 21% entertainment devices (such as television and radios), 8% refrigerators, and 4% power-saving lumps and chargers as their priority choice. Household members mainly need baking and cooking stoves to fulfill their basic needs, reduce costs of energy not only associated with an increasing biomass price but also with the cleanness or healthy nature of electricity and solar energy.

In terms of the frequency of stove use, 37% of electric users used electric stoves regularly while 21% used them only occasionally. The latter group used electric stoves sometimes due to a lack of adequate, reliable and affordable electricity supply and shortages, as well as the high price of stove technologies. To cope with these problems and cushion sudden electricity interruption, 28% were found to use traditional three-stone stoves always and 14% only sometimes. The absence of reliable supply and the use of biomass by households who have existing access to electricity, however, exacerbate increased deforestation and indoor air pollution.

### The use of ICS in informal settlements: the multinomial logit approach

This section presents various factors that have a potential impact on the choice of ICS in informal settlements. These factors were considered separately in the descriptive analysis. However, multinomial logit is used to evaluate the impact of those factors on households’ ICS use. It helps to estimate the direction and strength of the relationship between various factors and types of stoves households own. Before presenting the results of MLR, it is essential to describe the key variables considered in the model:

1. Sex: The gender of the household head selected for study is labeled as male or female.
2. Marital status: The marital status of the household head is labeled as single, married or separated (widowed and divorced).
3. Education level of the head of a household is categorized in four groups: Below grade 4, Grade 4-Grade 8, Grade 9-Diploma and Degree and above.
4. Family income: This is the monthly income generated by all family members and categorized into two groups: those earning up to 6000 *birr* and above 6000 *birr.*
5. Family size: This refers to the number of family members living in one home and grouped as up to 2, 3–4 and more than 4 families.
6. Years lived: From the first time a household owned the land, the number of years he/she lived in the area is categorized as those who lived up to 3, 4–6, 7–9, and more than 9 years.
7. Shelter type and condition: This is the material from which the house is made and its current condition labeled as poor (made from wood and mud), good (made from wood and cement) and very good (made from steel and blockets).
8. Shelter size: This refers to the number of quarters (rooms) in a home that can be described by the number of bedrooms and labeled as 1 room, 2 rooms, 3 rooms, and more than 3 rooms.
9. Land title: This is how informal settlers’ owned land is classified as purchased land, informally owned land and inherited land.
10. Factors affecting the choice of stoves: These factors include lowering cost of energy, saving family labor and time, availability, durability/quality, affordability, simplicity of use, safe and clean, subsidized and easy to obtain credit facilities, and stove recognition. The stoves are labeled as open fire three-stone stoves, *Mirt* stoves, *Lakech* stoves, and electric stoves.

Table 4 presents the parametric estimates used to determine households’ ICS choice and the estimated p-values. Then, households’ choice of ICS is measured by comparing each group of stoves (*Mirt stoves, Lakech stoves and electric stoves*) against the reference category (i.e., the *three-stone traditional stoves*) along a spectrum of variables. The reference category is used as a relative measure. Then, each group of stoves is compared to the base category centered on the relevant variables considered.

**Table 4:** Factors affecting the choice of energy-efficient stoves: the multinomial logit model

	Number of obs		=		423
	LR chi^2^(54)		=		388.51
	Prob > chi^2^		=		0.0000
	Pseudo *R*^2^		=		0.3491
3-stone stove	Coef.		Std. Err.	*Z*	*P* > /*Z*/
	(base outcome)				
*Mirt* stove					
Sex	0.0178	0.3567		0.05	0.09
Marital status	− 0.0382	0.3196		− 0.12	0.905
Education	0.2913	0.1786		1.63	0.105
Family income	− 0.4817	0.4002		− 1.20	0.229
Family size	− 0.1061	0.2696		− 0.39	0.694
Years lived	0.1674	0.1932		0.87	0.386
Shelter type	0.7682**	0.3508		**2.19**	0.029
Shelter size	0.2308	0.2215		1.04	0.297
Land title	0.0344	0.1944		0.18	0.859
Stove operating cost	− 0.2942**	0.1548		− **1.90**	0.057
Save labor and time	− 0.0721	0.1917		− 0.38	0.707
Availability of stove	− 0.3447***	0.187		− **1.84**	0.065
Quality of stove	− 0.2285	0.1664		− 1.37	0.17
Cost of stove	− 0.1489	0.2324		− 0.64	0.522
Simple to use	0.2853***	0.1661		**1.72**	0.086
Clean and safe stove	− 0.3426	0.2527		− 1.36	0.175
Subsidized stove	− 0.2064	0.1613		− 1.28	0.201
Widely known stove	− 0.3183**	0.1487		− **2.14**	0.032
_cons	2.1665	1.6167		1.34	0.18
*Lakech* stove					
Sex	− 0.3091	0.424		− 0.73	0.466
Marital status	− 0.0348	0.3733		− 0.09	0.926
Education	0.0455	0.2058		0.22	0.825
Family income	− 0.6546	0.4644		− 1.41	0.159
Family size	− 0.1019	0.3128		− 0.33	0.744
Years lived	0.0915	0.2164		0.42	0.672
Shelter type	1.1119*	0.4074		**2.73**	0.006
Shelter size	0.0951	0.2529		0.38	0.707
Land title	− 0.4505***	0.254		− **1.77**	0.076
Stove operating cost	0.0348	0.1823		0.19	0.848
Save labor and time	− 0.1512	0.2312		− 0.65	0.513
Availability of stove	0.0348	0.1933		0.18	0.857
Quality of stove	− 0.0394	0.1964		− 0.20	0.841
Cost of stove	0.1643	0.2418		0.68	0.497
Simple to use	0.074	0.1894		0.39	0.697
Clean and safe stove	0.0242	0.3177		0.08	0.939
Subsidized stove	− 0.1756	0.1828		− 0.96	0.337
Widely known stove	0.0476	0.1787		0.27	0.79
_cons	− 0.7658	1.8939		− 0.40	0.686
Electric stove					
Sex	0.3383	0.4269		0.79	0.428
Marital status	0.5568	0.4766		1.19	0.234
Education	1.0173*	0.2694		**3.78**	0
Family income	0.2463	0.5561		− 0.44	0.658
Family size	− 0.2987	0.3284		− 0.91	0.363
Years lived	0.6742*	0.238		**2.83**	0.005
Shelter type	1.2905*	0.4482		**2.88**	0.004
Shelter size	0.3646	0.2768		1.32	0.188
Land title	0.2652	0.2401		1.10	0.269
Stove operating cost	1.4825*	0.2684		**5.52**	0.000
Save labor and time	0.7222	0.4486		1.61	0.107
Availability of stove	− 0.2224	0.2045		− 1.09	0.277
Quality of stove	0.7756*	0.2235		3.47	0.001
Cost of stove	− 0.7326*	0.294		− **2.49**	0.013
Simple to use	0.8286*	0.2988		**2.77**	0.006
Clean and safe stove	− 0.3461	0.4797		− 0.72	0.471
Subsidized stove	− 0.3315	0.2000		− 1.66	0.097
Widely known stove	0.2396	0.2054		1.17	0.243
_cons	− 19.5514	3.2376		− 6.04	0.000

Note: *, ** and *** are statistically significant at *p* < 1%, *p* < 5% and *p* < 10%, respectively and the bold figures inside the table indicate the significant variables affecting households stove choice

Source: Data developed by the author, Feb., 2022

Valid households considered for this analysis are 423 (94%) and the Chi-square test indicating the model with those factors considered, significantly affects the choice of ICS with χ^2^(54) = 388.51 and *p* < 0.0000. Although a higher value of Pseudo R2 closer to one indicates the best fit of the model, the outcomes of this study are still valid.

The analysis indicated that provided all other relevant variables in the model remain constant, relative to the traditional three-stone stove, as households’ shelter condition improves by one unit (i.e., from poor to good or from good to very good conditions), the number of households using *Mirt* stoves increases by 0.7678 units; as the stove-operating cost increases by a unit, its demand decreases by 0.2942 units compared to prior periods; if its availability decreases in the market or when the level of shortage increases by a unit, its demand decreases by 0.3447 units; as the simplicity to use the stove increases by one level, households’ demand increases by 0.2853; as the households lack of awareness/recognition level increases by a unit, the demand for *Mirt stoves* decreases by 0.3183 units.

The choice of *Lakech* stoves depends on shelter type and land title held by a household. Like *Mirt* stoves, under ceteris paribus assumption, as the shelter condition owned by a household improves by one level, the number of *Lakech* users increases by 1.1119 units relative to the three-stone traditional stove users. That is, household members who lived in a relatively good shelter use more *Lakech* stoves than the open fire traditional three-stone stoves. However, as informal settlers’ land insecurity increases by one unit and if there is a high possibility of eviction by a single letter, the number of *Lakech* users decreases by 0.4505 units while those using applying the traditional three-stone stoves increases.

The choice of electrical stoves is influenced by various factors. The data show that household heads with higher levels of education who live in relatively better homes (such as those who live in good and very good shelter) and remain for longer periods in the area, are also found to be using more electrical stoves than the traditional three-stone stoves. For instance, holding all other relevant variables constant, as household heads education increase by one level, the number of electric stove users increases by 1.0173 units; as the length of time a household lived in the area increases by 1-year, electric stove users increase by 0.6742 units; and as the condition of shelter owned by residents improves by one stage, the demand for electrical stoves increases by 1.2905 units relative to the traditional three-stone users.

Household members also choose electrical stoves that lower the operating cost of energy (save family labor and time), and that are long-lasting, affordable, and simple to use. For example, under ceteris paribus assumption and relative to the three-stone stoves, as electrical stoves save operating cost and this saving increases by one unit, the demand for this stoves increases by 1.4825; as the quality of electrical stoves increases by one unit (become more durable), their demand increases by 0.7756; and as the purchase price of these stoves increases by one unit, their demand decreases by 0.7326; and as the simplicity to use electrical stoves improves by one unit, their demand increases by 0.8286.

Similarly, household heads with higher levels of education living in relatively better homes for longer periods in an area are found to be using more electrical stoves than the traditional three-stone stoves. The empirical data show that, holding all other relevant variables constant, as household heads education increase by one level, the number of electric stove users increase by 1.0173; as the condition of shelter owned by residents improves by one unit, the demand for electrical stoves increases by 1.2905; and as the length of time a household lived in an area increases by one year, electric stove users increase by 0.6742 relative to the traditional three-stone users.

Household members also choose electrical stoves that are of high quality /long lasting/, affordable, and simple to use. Such stoves are found to lower the operating cost of energy and save family labor and time. For example, under ceteris paribus assumption, as the quality or durability of electrical stoves increases by one unit, the number of households using them increases by 0.7756. As the purchasing cost of the stove increases by one unit (i.e., if the price becomes more expensive), its demand decreases by 0.7326 units; and an increase in electricity tariffs by one unit leads to an increase in electrical stove usage by 1.4825 units. The latter result could signify, in addition to other benefits, that electricity is clean and more convenient to use, and the tariff is still lower than the cost of employing biomass. However, these stoves are less affordable and less subsidized than the traditional three-stone stoves.

The findings from the descriptive analysis and the MLR model yielded similar results for many of the variables considered. In both cases, the factors affecting the choice of stove vary based on the type of stove considered. However, the descriptive analysis shows the positive contribution of shelter size to own ICS while this is not a significant factor in the logistic regression. This is because the descriptive analysis treated the factors affecting the choice of stoves independently while the MLR estimated the interactive effect of all variables on households’ energy-efficient stove choice.

## Conclusions and implications

### Conclusions

ICS burn fuels cleanly, save time, cost, workload of family members involved in cooking activities, and contribute to the sustainable usage of scarce resources. The choice of these stoves and appliances, however, depend on households’ socio-economic backgrounds and availability as well as affordability of alternative energy sources.

The findings of both MLR and descriptive statistics showed that sex, family size and family income have no relationship with the usage of energy-efficient stoves. However, education level of the household head, the number of years lived in the area, shelter type and size owned, energy price /operating cost/, availability, affordability and quality of the stove have significant influence on ICS usage in informal settlements. These technologies help to meet ever-increasing energy needs of households and save expenditures for energy while protecting the environment.

At present, due to a shortage of fuel wood and lack of access to reliable electricity supply in Ethiopia, households using ICS are growing significantly in number (8% before 10 years to 51% in the last 5 years).

### Implications

Since the prices of ICS are rapidly increasing, arranging loan facilities, flexible payment systems, subsidies and incentives to households are critical policy issues. On the other hand, such schemes should discourage the use of open fire traditional stoves and the consumption of high polluting energy sources through requiring payments in the form of taxes and penalties. Solar energy should also be used beyond lighting and charging of batteries. Integrating households’ socio-economic backgrounds with their energy sources promoting the usage of energy-efficient cooking stoves could be seen as a major policy issue in informal settlements.

### Future research directions

This study analyzed the factors that determine the adoption of ICS in informal settlements from the perspectives of households using cross-sectional data. Future research, however, could focus on ICS suppliers’ problems, government policies and energy consumption behavior of household members by using panel data and optimizing the needs of different stakeholders.

## Data Availability

The data that supported the findings of this study were generated from households in informal settlements by using questionnaires and interviews. It is placed in the text and hyperlinked with the manuscript. Other data (when needed) can be obtained on request from the corresponding author.

## Abbreviations

CFL: Compact fluorescent lamps
EET: Energy-efficient technologies
ICS: Improved cook stoves
LED: Light-emitting diodes
LPG: Liquefied petroleum gas
MTF: Multi-tier framework
PV: Photovoltaic
SDGs: Sustainable development goals

## Footnotes

1 *Injera* is flatbread traditional staple food made from fine iron-rich *Teff* (agricultural product typically grown in Ethiopia) sometimes mixed with wheat, barley or sorghum flour.

2 *Mitad* is a three-stone traditional stove used to bake *Injera* (staple food in Ethiopia) using fuel wood, plant residues and animal dung.

3 *Fermelo* is a stove used to cook dishes using charcoal in an open fire.

4 Electric users are households using electric power for cooking and baking in addition to lighting. They obtained electric power from the Ethiopian Electricity Utility legally or from their neighbor by sharing electric cost.

5 Non-users of electricity refer to households who either use electric power from their neighbors only for illumination purpose or those who do not use it at all.
